# Far Anterior Medial Portals in Complicated Elbow Arthroscopic Procedures: Safety Profile in a Cadaveric Model

**DOI:** 10.1016/j.asmr.2021.11.009

**Published:** 2021-12-30

**Authors:** Leland C. McCluskey, Tucker J. Cushing, John M. Weldy, Nisha N. Kale, Felix H. Savoie, Gleb Medvedev

**Affiliations:** Department of Orthopaedic Surgery, Tulane University, New Orleans, Louisiana, U.S.A.

**Keywords:** arthroscopy, elbow, elbow portals, anatomy

## Abstract

**Purpose:**

The purpose of this study is to describe the placement and evaluate the safety of the far anterior proximal and distal anteromedial portals by comparing them to previously defined portal techniques in a cadaveric model of the elbow.

**Methods:**

Six paired (left and right) fresh, frozen cadaveric elbow joints were dissected. .62-mm Kirschner wires were placed at the literature-defined distal and proximal portal sites on right elbows. The proposed “far anterior” distal and proximal portals were established on the matched left elbows. The elbows were dissected to display the median and ulnar nerves. Digital calipers were used to measure distances from wires to nerves.

**Results:**

For the distal portal, the literature-defined portals were a significantly greater distance (*P* = .014) from the ulnar nerve (31.22 mm) compared to the far anterior portals (24.65 mm). For the proximal portal, the far anterior portals were a significantly greater distance (*P* = .026) from the ulnar nerve (26.98 mm) than the literature-defined portals (13.75 mm). There was no significant difference between the far anterior and literature-defined proximal and distal portal techniques in relation to the median nerve.

**Conclusions:**

Analysis of elbow arthroscopy anteromedial portal technique shows the far, anterior, proximal, and distal portals are a safe distance from the ulnar and median nerves. A portal modification that may address complicated elbow conditions is a more anterior placement of the medial portals to allow for better visualization and access.

**Clinical Relevance:**

The elbow is a difficult joint in which to perform arthroscopic surgery. One option our institution has used for safe portal modification to address complicated elbow conditions is a further anterior placement of the medial portals to allow better visualization and access.

## Introduction

Correct portal placement is a key step in elbow arthroscopy, as variations from known portal locations can not only make the procedure very difficult, but also place neurovascular structures at risk.^1^ In recent years, arthroscopic surgery of the elbow has progressed to include multiple approaches and technical variations to treat a wider variety of elbow pathology than originally thought possible.^2^^,^^3^ Optimizing portal placement allows for improved visualization and instrument placement to better view and treat different anatomic areas and pathology.^4^, ^5^, ^6^ Complicated procedures such at ankylosis takedown, removal of arthritic spurs, fracture fixation, excision of heterotopic ossification, and many others may require specialized portals for satisfactory completion of the procedure.

**Fig 1:**
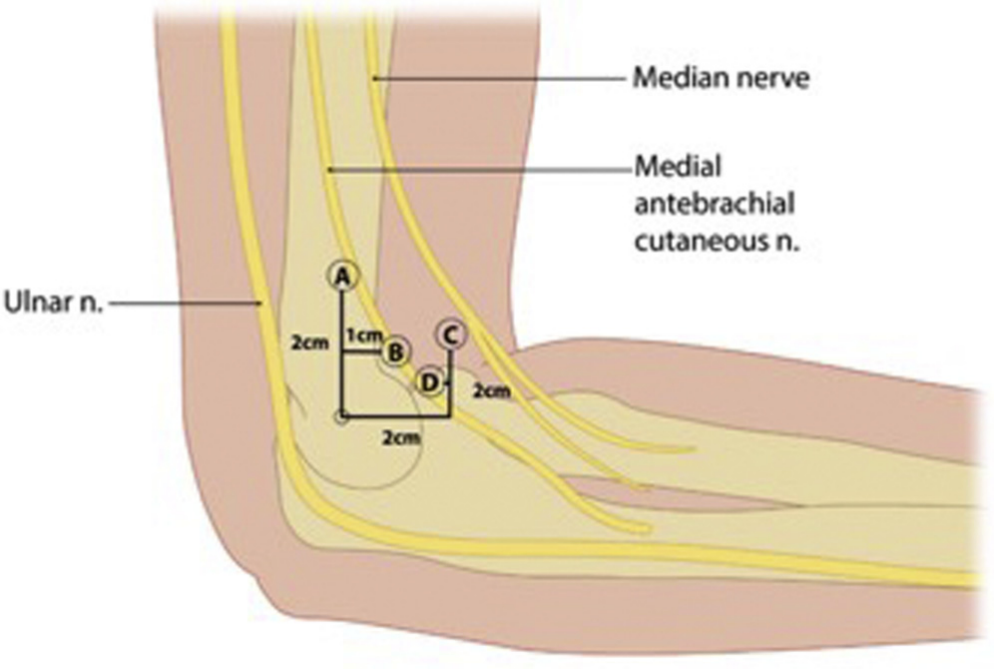
Described locations of medial elbow arthroscopy portals. Proximal portals: (A) 2 cm proximal, just anterior to intermuscular septum (Poehling et al)^17^, 2 cm proximal (Chaware et al.).^14^ (B) 2 cm proximal, 1 cm anterior (Lindenfield et al.).^9^ Distal portals: (C) 2 cm distal, 2 cm anterior (Andrews et al.).^3^ (D) 2 cm distal, 1 cm anterior (Verhaar et al.).^10^

The first anteromedial portal location was described by Andrews and Carson in 1985.^7^ Since then, other medial elbow arthroscopy portals have been described and grouped broadly into proximal and distal, based on location relative to the medial epicondyle. There have been at least five separate definitions reflecting distal and proximal anteromedial portal locations (Fig 1).^7^, ^8^, ^9^, ^10^, ^11^, ^12^, ^13^, ^14^, ^15^, ^16^, ^17^ Few studies have deviated from these definitions.

On the basis of practices at our institution, we have modified the existing proximal and distal portal techniques to a more anterior location to facilitate access for both instrumentation and visualization during advanced surgical procedures. We term these portals as far anterior proximal and distal.

Portal placement safety in regard to neurovascular anatomy is well studied in cadaveric models.^7^, ^8^, ^9^, ^10^, ^11^, ^12^, ^13^, ^14^, ^15^, ^16^ Proximal portal placement was originally described by Poehling et al. in 1989 as 2 cm proximal the medial epicondyle and just anterior to the intermuscular septum (Fig 1).^17^ Literature review demonstrated Poehling et al.’s portal placement as the most studied proximal portal. Therefore, it was chosen as the standard for the literature-defined proximal portal location in this study.^18^ The far anterior proximal portal is located 2.5 cm proximal and 3 cm anterior to the medial epicondyle. Distal portal placement was originally described by Andrews and Carson as 2 cm distal and 2 cm anterior to the medial epicondyle, which is still commonly used today and is used in this study as the literature-defined distal portal.^7^ This portal was the most frequently studied portal in other cadaveric studies per Cushing et al.^18^ The far anterior distal portal is placed 2 cm anterior to the medial epicondyle.

Indications for elbow arthroscopy are expanding, creating the need to modify the originally described portals in order to obtain better visualization and functionality.^4^, ^5^, ^6^ It is the opinion of the senior author that far anterior portal placement is useful for advanced cases, including deformity due to arthritis, ankyloses, complicated instability, and acute fracture dislocations, as far anterior portal placement provides superior visualization and functionality. Using the new portal placement allows the surgeon to enter the elbow on the medial side of the joint rather than in the center and also is more protective of the anterior neurovascular structures as the more anterior starting point prevents medial deformity from forcing the cannula anteriorly during insertion. The purpose of this study is to describe the placement and evaluate the safety of the far anterior proximal and distal anteromedial portals by comparing them to previously defined portal techniques in a cadaveric model of the elbow. We hypothesize that the far anterior proximal and distal anteromedial portals provide a safety profile that is not inferior to that of literature-defined, universally accepted anteromedial portals.

## Methods

Six paired, left and right joints for a total of 12 fresh, frozen cadaveric elbow joints without forearms and hands were purchased from Science Care Phoenix, AZ, using departmental funds, and dissected and evaluated for data measurement and collection. Sample size was selected based on existing literature regarding portal cadaver studies.^18^ Specimens were assessed by faculty authors (F.S., G.M.). There was 7-8 cm of forearm remaining for reach specimen measured from the elbow crease. The humerus and forearm were secured to stimulate standard prone patient positioning, as described by Poehling et al.,^17^ with 90° of shoulder abduction and 90° of arm flexion with the arm hanging.

Each elbow was held at 90° of flexion and placed into a vice grip with the forearm pointing toward the ground, simulating prone patient positioning. The proximal ulna, medial epicondyle, and ulnar nerve were all identified by palpation and were marked using a surgical marking pen. Proximal and distal anteromedial portals were then marked using a ruler and marking pen. Each right elbow was used to analyze literature-defined anteromedial portals, and each left elbow was used to analyze the proposed far anterior portal technique.^7^^,^^17^ A single sports medicine-trained orthopaedic surgeon (F.S.) placed all of the literature-defined portals and another hand- and elbow-trained orthopaedic surgeon (G.M.) placed all of the far anterior portals in order to minimize variations in technique used between different specimens. The first author (L.M.) measured the pin-to-nerve distance. Far-anterior and literature-defined portals were placed on opposite sides due to the increased probability of measurement error and anatomical limitations of creating two portal incisions on a single specimen. The literature-defined proximal portal was placed at 2 cm proximal and slightly anterior to the intermuscular septum, as described by Poehling et al.^17^ The literature-defined distal portal was placed 2 cm distal and 2 cm anterior to the medial epicondyle, as described by Andrews and Carson.^7^ The far anterior proximal portal was placed 2.5 cm proximal and 3 cm anterior to the intermuscular septum. The far anterior distal portal was placed 2 cm anterior to the medial epicondyle (Table 1).

**Table 1:** Anteromedial Portal Placement as Used in This Study

Proximal		Distal	
Literature-Defined	Far Anterior	Literature-Defined	Far Anterior
2 cm proximal ×Just anterior to intermuscular septum	2.5 cm proximal × 3 cm anterior to intermuscular septum	2 cm distal × 2 cm anterior to medial epicondyle	2 cm anterior to medial epicondyle

**Fig 2:**
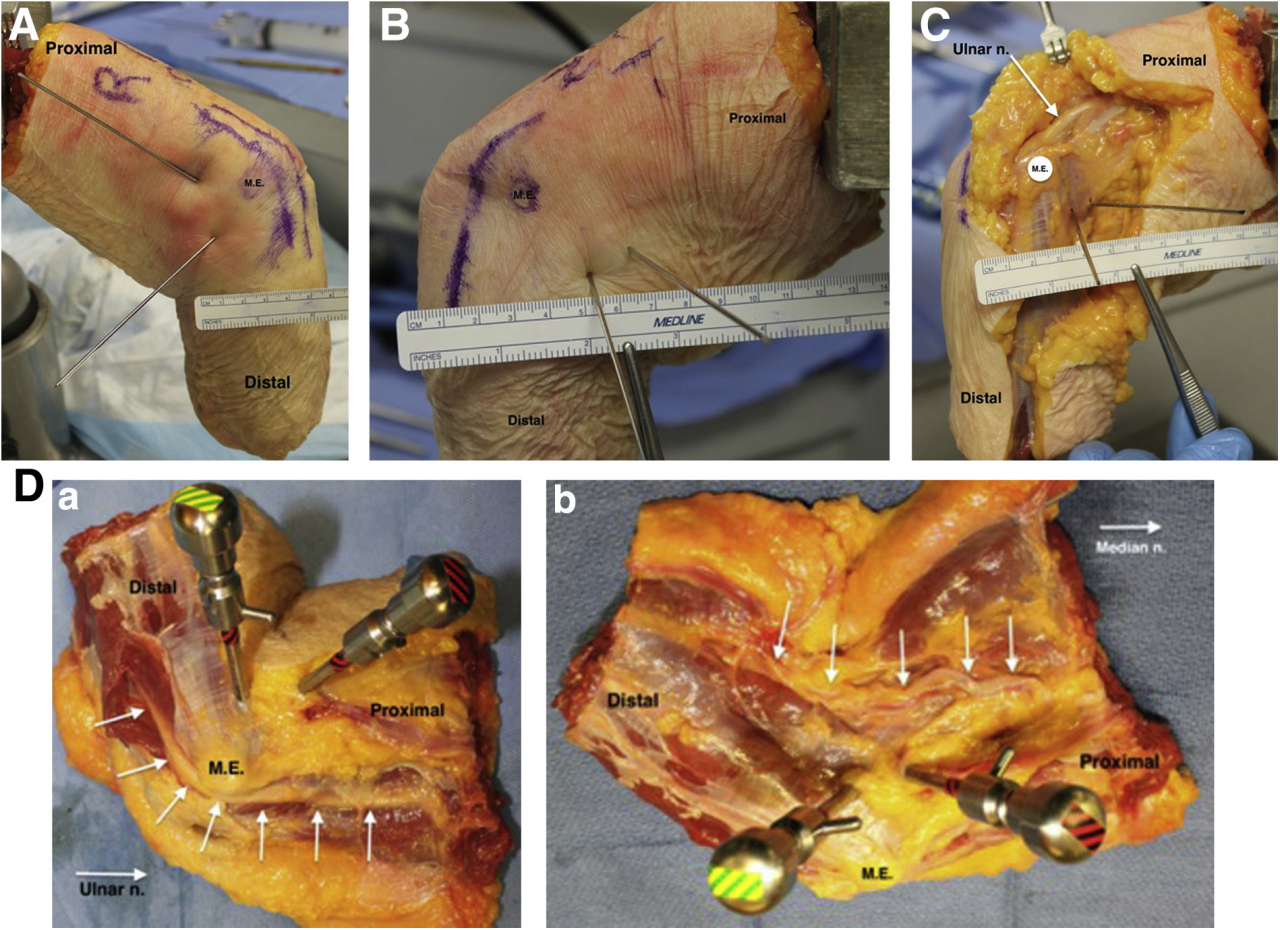
Methods. (A) Photograph of a right elbow demonstrating literature-defined distal and proximal portal placement. Medial epicondyle and course of ulnar nerve marked with skin marker. The ruler shows the far anterior distal portal 2cm from the medial epicondyle if spatially oriented as anterior from the medial epicondyle. (B) Photograph of a left elbow demonstrating far-anterior distal and proximal portal placement. Medial epicondyle (labeled M.E.) and course of ulnar nerve marked with skin marker. The anterior portal wire is the one farthest to the far anterior one. The ruler shows ∼2.5 cm if spatially oriented as anterior from the medial epicondyle. (C) Specimen B after dissection of skin and subcutaneous tissue with ulnar nerve identified. The anterior portal wire is the one furthest to the far anterior one. (D) Left elbow with arthroscopy trochars placed to demonstrate the surgical portals as they would most likely exist in the operating room. Top: Medial epicondyle and ulnar nerve are identified. Bottom: Medial epicondyle and median nerve are identified.

Joint insufflation was accomplished using 30 cc of water or until significant resistance was met on the syringe plunger, as previously described in similar cadaveric studies.^9^^,^^11^^,^^13^^,^^14^^,^^15^ Then, .62 mm Kirschner wires were placed by hand through the marked portal sites into the elbow joint. Dissection was performed until the ulnar and median nerves were identified. The closest possible line connecting nerves and K-Wires was measured for each specimen using Vernier 150-mm Digital Calipers, accurate to .01 mm. Figure 2 demonstrates elbow positioning, K-wire placement, and dissection of specimens.

One measurement was made for reach respective portal (including both literature-defined portals and far anterior locations for the proximal and distal portals for the ulnar and median nerve). Measurements and statistical analysis, including unpaired *t*-tests, were run to determine whether there was a difference in measurement between the literature-defined and far anterior locations for proximal and distal portals for the ulnar and median nerve, respectively. Additionally, the results were compared to other results gathered in a recently published systematic review of this topic.^18^ Statistical analysis was performed using with SPSS Statistics 21 (IMB, Armonk, New York). A *P* value of < .05 was considered statistically significant.

## Results

**Fig 3:**
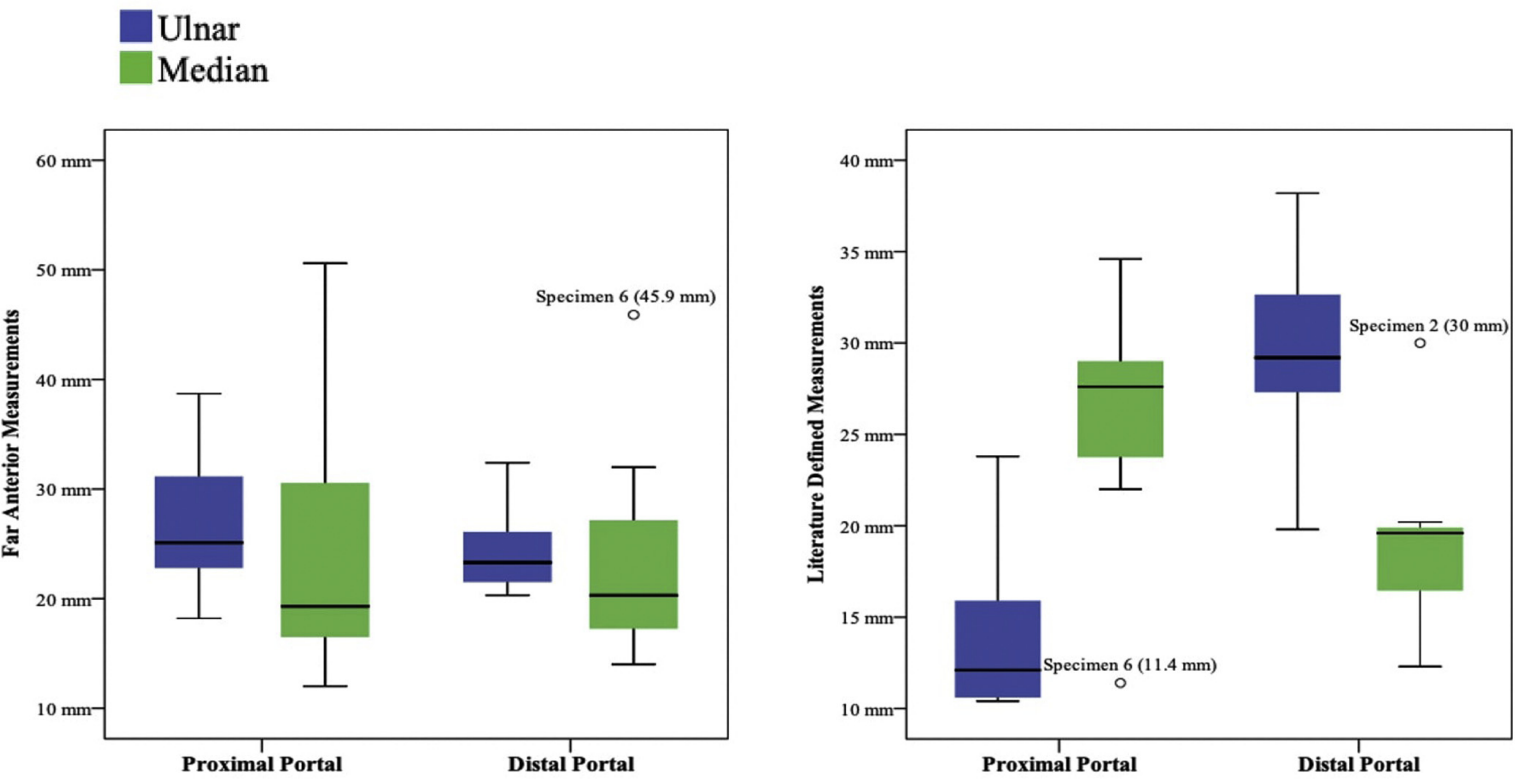
Box plot of cadaver specimen portal measurements, including outlier for specimen 6.

The data and measurements from our cadaver dissections are organized in Tables 2 and 3. Discrete measurements from the six specimens are presented in Table 2. Specimen 6 was determined to be an outlier, as assessed by averages and inspection of a boxplot for values greater than 1.5 box lengths from the edge of the box and was eliminated from analysis (Fig 3). Data are indicated as means ± SD, unless otherwise stated. Measurements for the literature-defined and far anterior techniques for the proximal and distal portals with respect to the distance from the ulnar and median nerve were normally distributed, as assessed by Shapiro-Wilk’s test (*P* > .05).

**Table 2:** Discrete Data of Proximal and Distal Measurements From Ulnar and Median Nerves For Cadaver Specimens

Distance From Nerve	Specimen	Proximal Portal		Distal Portal	
		Literature Defined (mm)	Far Anterior (mm)	Literature Defined (mm)	Far Anterior (mm)
Ulnar	1	12.1	38.7	38.2	32.4
	2	23.8	25.1	33.4	20.3
	3	10.6	21	31.9	22.2
	4	10.4	24.6	29.2	26
	5	15	18.2	27.3	20.8
	6∗	16.8	28	19.8	23.3
	7	10.6	34.3	27.3	26.2
Median	1	27.6	36.1	19.6	19.3
	2	34.6	25	30	20.3
	3	25.5	12	14.4	15.2
	4	28.5	16.8	19.6	22.3
	5	22	16.2	18.5	14
	6∗	11.4	50.6	12.3	45.9
	7	29.5	19.3	20.2	32

∗ Specimen 6 was determined to be an outlier as assessed by averages and inspection of a boxplot for values greater than 1.5 box lengths from the edge of the box, and was eliminated from analysis

**Table 3:** Descriptive Measurements of Proximal and Distal Portals

Distance From Nerve	Descriptive Statistics	Proximal Portal		Distal Portal	
		Literature Defined	Far Anterior	Literature Defined	Far Anterior
Ulnar	Average	13.75 mm	26.98 mm	31.22 mm	24.65 mm
	SD	5.22 mm	7.91 mm	4.21 mm	4.56 mm
	Variance	27.27 mm^2^	62.60 mm^2^	17.75 mm^2^	20.81mm^2^
	Range	13.4 mm	20.5 mm	10.9 mm	12.1 mm
Median	Average	27.95 mm	20.90 mm	20.38 mm	20.52 mm
	SD	4.21 mm	8.59 mm	5.16 mm	6.44 mm
	Variance	17.69 mm^2^	73.70 mm^2^	26.62 mm^2^	41.46 mm^2^
	Range	12.6 mm	24.1 mm	15.6 mm	18.0 mm

For the distal portal, the literature-defined portal measurements (31.22 ± 4.21 mm) were a greater distance from the ulnar nerve compared to the far anterior portal measurements (24.65 ± 4.56 mm), a distance that was statistically significant (95% confidence interval [CI], 2.01 to 11.12), *t*(5) = 3.704, *P* = .014). For the proximal portal, the far anterior portal measurements (26.98 ± 7.91 mm) were a greater distance from the ulnar nerve than the literature-defined measurements (13.75 ± 5.22 mm), a distance that was statistically significant (95% CI, −24.14 to −2.32), *t*(5) = −3.12, *P* = .026. There was not a statistically significant difference between literature-defined and far anterior measurements for the proximal (*P* = .966) or distal (*P* = .084) portals for the median nerve (Table 4).

**Table 4:** Independent Samples *t*-Test

Distance From Nerve	Proximal Portal			Distal Portal		
	Literature Defined (mm)	Far Anterior (mm)	*t*-Test	Literature Defined (mm)	Far Anterior (mm)	*t*-Test
Ulnar	13.75 (5.22)	26.98 (7.91)	.026	31.22 (4.21)	24.65 (4.56)	.014
Median	27.95 (4.21)	20.90 (8.58)	.084	20.38 (5.16)	20.52 (6.44)	.966

Measurements are listed as means (SD).

The data from the systematic review by Cushing et al. is summarized in Table 5.

**Table 5:** Results from Systematic Review by Cushing et al.

Study	Average Distance to Ulnar Nerve (if reported) (mm)		Average Distance to Median Nerve (if reported) (mm)		Average Distance to Brachial Artery (if reported) (mm)		Average Distance to MABCN (if reported) (mm)	
	Distal	Proximal	Distal	Proximal	Distal	Proximal	Distal	Proximal
Andrews and Carson (w/o)^7^			6					
Lynch et al (w and w/o)^8^			4 (range 3-10mm) (w/o), 14 (w)		9 (range 8-13 mm) (w/o), 17 (w)		1 (range 1-9 mm) (w)	
Lindenfeld (w)^9^		23.7 ± 1.63		22.3 ± 1.63				
Verhaar et al (w/o)^10^	> 25		18 (range 12-25mm)		26 (range 21-32 mm)			
Adolfsson et al (w)^11^	24 (range 19-26 mm)	21 (range 19-24 mm)	14 (range 11-18 mm)	19 (range 16-22 mm)	22∗∗		4 (range 2-11 mm)	6 (range 3-12 mm)
Stothers et al (w/o)^12^		12 (range 7-18 mm)	7 (range 5-13 mm)	12.4 (range 7-20 mm)	15.2 (8-20 mm)	18 (12-25 mm)	1 (range 0-5 mm)	2.3 (range 0-9 mm)
Unlu et al∗ (w)^13^	25.4 ± 1.7	20 ± 2.2	16.2 ± 4.4	17.1 ± 2.8	20.3 ± 3.4	21.1 ± 3.3	9.2 ± 3.9	7.9 ± 2.5
Chaware et al (w)^14^	16.03 ± 4.86	13.16 ± 3.73	22.12 ± 6.64	19.45 ± 7.42			4.99 ± 5.03	5.14 ± 5.08
Marshall et al (w/o)^16^			6.5 ± 3.3					
Zonno et al (w)^15^		20.8 (range 14.4-25.1 mm)						
Average distance and standard deviation (All Studies)	22.61 ± 4.42	18.44 ± 4.72	12.98 ± 5.95	18.05 ± 3.67	20.1 ± 4.24	19.55 ± 2.19	4.04 ± 3.39	5.34 ± 2.33
Average distance and standard deviation (w/ joint distension)	21.81 ± 5.05	19.73 ± 4.28	16.58 ± 3.84	19.46 ± 2.15	19.77 ± 2.54	21.1 ± 0	4.8 ± 3.39	6.35 ± 1.41
Average distance (w/o joint distension)	25 ± 0	12 ± 0	8.3 ± 5.54	12.4 ± 0	16.73 ± 8.6	18 ± 0	1 ± 0	2.3 ± 0

The average measured distances to the ulnar nerve, medial nerve, brachial artery, and median antebrachial cutaneous nerve (MABCN) reported at each portal site for each study. The average distances and associated standard deviations from neurovascular structure to portal site between all studies, studies that only included joint distension, and studies that did not include joint distension. Standard deviations (reported in table as ± SD) and ranges of measurements reported in each study were included, if available. Of note, the values reported in the table are with the elbow in 90° of flexion wherever applicable to standardize comparison. w/o, measured without joint distention; w, measured with joint distention. There was no standard deviation^8^ or range to report from the Andrews and Carlson^3^ study.

MABCN, medial antebrachial cutaneous nerve; w, measured with joint distention; w/o, measured without joint distention.

∗ Best results from the study in full flexion and neutral pronation/supination rotation.^9^

∗∗ Reported as 8mm further from the mean median nerve distance (14 mm) to the distal portal, which therefore was assumed to equal 22 mm.^7^

## Discussion

Our results found that there is a statistically significant improved safety margin to the ulnar nerve when using the far anterior proximal portal. Portal locations are vital to attain adequate visualization within the elbow joint, but they must have an adequate safety profile to minimize the risk of iatrogenic neurovascular injury. In our opinion, the most common error in medial portal placement is to place it too close to the intermuscular septum. This not only increases risk of ulnar nerve injury but also makes joint entry more difficult. In advanced cases, such as arthritic spur removal and fracture dislocation, the deformity on the medial side will direct the cannula anteriorly into the area of the median nerve and brachial artery. The far anterior portal is not only safer in relation to the ulnar nerve, but it allows an easier and more medial entry into the elbow, creating the improved visualization and surgical instrumentation angles, which are needed for advanced arthroscopic procedures. This article seeks to describe the location of our institutional portals and demonstrate their safety to other surgeons.

The elbow is a complex joint with several neurovascular structures located in a relatively small area.^1^ The brachial artery, median nerve, ulnar nerve, and radial nerve all pass through the elbow and are at-risk structures during an arthroscopic approach to the elbow joint.^19^^,^^20^ The median and ulnar nerves are the major structures at risk when establishing medial portals.^21^^,^^22^ Because of the density of important structures in this area, safety of these portals is relative, meaning that distances are more aptly described in terms of millimeters, and there is little room for error.

Our results reveal that there is a statistically significant improved safety margin to the ulnar nerve when using our far anterior proximal portal of 2.5 cm proximal and 3 cm anterior to the medial epicondyle, compared to literature-defined portals of 2 cm proximal and just anterior to the intermuscular septum.^17^ For years, surgeons have been placing the proximal portal just anterior to the intermuscular septum with known risk to the ulnar nerve;^23^^,^^24^ thus, the improved safety of the far anterior portal is an expected benefit, given the known course of the ulnar nerve.

There was no statistically significant difference appreciated between the far anterior proximal portal and the literature-defined counterpart when observing proximity to the median nerve. However, the distance averaged 20.90 ± 8.59 mm, suggesting that clinically, the proximal far anterior portal is a safe distance from the median nerve, despite the lack of statistical significance.^18^^,^^19^ This conclusion is based on comparison of our data to the data collected in the systematic review by Cushing et al.^18^ The two studies included in this review specifically looked at the median nerve distance from the proximal portal described by Poehling et al, which used the literature-defined portal in this study, and they showed the average distance to the median nerve to be 12.4 mm (range 7-20 mm)^12^ and 19.45±7.42 mm.^14^ Lindenfeld et al. found that a distal portal is closer to the distal elbow capsule, and advancing a cannula straight medially toward the median nerve puts it at an increased risk of injury. Proximal portals allow the surgical cannula to be inserted distal and parallel to the median nerve, decreasing the risk of injury.^9^ This leads us to the conclusion that the safety profile of the far anterior proximal portal is acceptable with regard to the median nerve. In addition, when dealing with significant medial deformity, the spurs along the medial side will often direct the canula in a more anterior direction during insertion, increasing the risk of median nerve or brachial artery injury.^25^

The literature-defined distal portal of 2 cm distal and 2 cm anterior to the medial epicondyle demonstrated a statistically significant increase in distance to the ulnar nerve when compared to our far anterior distal portal of 2 cm anterior to the medial epicondyle with no distal projection.^7^ Because there is no distal projection of the far anterior portal, closer proximity to the ulnar nerve is plausible. Although there was a statistically significant difference that seems to demonstrate improved safety of the literature-defined distal portal, this result is not clinically significant given that the distances measured from our far anterior distal portal to the ulnar nerve are >2 cm (24.65 ± 4.56 mm), which has been described as an acceptable safe distance from neurovascular structures.^18^^,^^19^ Additionally, when we compare our results for distal portal safety to the results in the literature, our results show that our far anterior distal portal averaged a greater distance from the ulnar nerve when compared to previous studies,^7^^,^^8^^,^^11^^,^^8^^,^^12^^,^^16^ except for the study by Unlu et al.,^13^ which averaged 25.4 ± 1.7 mm to the ulnar nerve, a difference of only 2 mm.

With regard to the median nerve, there was again no statistically significant difference appreciated between the far anterior distal portal and its literature-defined counterpart. This again does not allow us to draw definitive conclusions as to the safety when comparing the two portal placements. However, when we compare our results for distal portal safety to the results in the literature, our results show that our far anterior distal portal averaged a greater distance from the median nerve (20.52 ± 6.44 mm) compared to previous studies,^7^^,^^8^^,^^11^^,^^12^^,^^16^ except for Chaware et al.,^10^ which averaged 22.12 ± 6.64 mm to the median nerve. Again, we consider this difference of <2 mm to be clinically insignificant. These results demonstrate that our far anterior distal portal achieves at least an equal safety profile when compared to safety margins in the current literature.

Overall, our data most strongly support the safety margin of the far anterior proximal portal with regard to the ulnar nerve, as this was the only set of measurements that achieved a statistically significant improved safety margin. The safety margin of the far anterior distal portal with regard to the ulnar nerve is also acceptable based on the fact that these values are all greater than 2 cm. The safety margins of both portals with regard to the median nerve do not have statistically conclusive data; however, we believe these margins are clinically safe on the basis of comparisons to the measurements reported in the systematic review by Cushing et al.,^18^ which show that our far anterior proximal and distal portal measurements to the median nerve match or exceed the safety profiles of literature-defined portals that are widely accepted and used commonly in current clinical practice.

The key point of the far anterior portals is to improve safety during advanced procedures, especially arthritic spur excision and fracture management. The far anterior portals have an acceptable safety margin in normal elbows, but in our opinion are critical in deformed elbows to increase the safety of the procedures. These more anterior portals allow the surgeon to work around the deformity and more easily excise the spurs or align fracture fixation while maintaining a safe margin around both the ulnar nerve and the anterior neurovascular structures.

Future studies related to alternative portal techniques may include clinical studies rather than cadaveric ones. Other studies may include supine versus prone positioning to determine gravity dependence of patient positioning. Additional variables, such as the effect of joint distension and pronation versus supination of the forearm also need to be explored. Additionally, a study that objectively demonstrates improved visualization is obtained through our far anterior portals would further support the proposed technique by providing clear, objective clinical superiority when compared to the traditional, literature-defined portal placement.

### Limitations

This study does have limitations. Because our cadavers were devoid of hands, forearms and shoulder tissue, the lack of gravitational pull by this devoid material may skew the measurements between portals and nerves. This study does not investigate distances to other neurovascular structures in range of our portals, such as the brachial artery, medial antebrachial cutaneous nerve, and vascular structures, so describing safety margin is only limited to the ulnar and median nerves. Because we conducted this study using cadavers, reduced elasticity of the cadaveric tissue could negatively impact the effects of joint distension in providing visualization and space between portal and nerves.^16^ We eliminated one pair of cadaver elbows in this study because some of the data collected from this specimen made it an obvious outlier during our statistical analysis, being far from the measurement range that we observed in the other specimens (Fig 3). This aberrant data could be due to previous trauma, ulnar nerve transposition, or misidentification of structures during dissection. Finally, data about cadaver specimens, including age, weight, and gender, were not available for our purchased specimens. The size of the patient could also impact the distance from portal to nerve, and a strict millimeter measurement may not necessarily apply to a morbidly obese or very petite patient.

### Conclusions

Analysis of elbow arthroscopy anteromedial portal technique shows the far, anterior, proximal, and distal portals are a safe distance from the ulnar and median nerves. A portal modification that may address complicated elbow conditions is a more anterior placement of the medial portals to allow for better visualization and access.

## Supplementary Data

ICMJE author disclosure forms
